# Relative Hypo- and Hypercortisolism Are Both Associated with Depression and Lower Quality of Life in Bipolar Disorder: A Cross-Sectional Study

**DOI:** 10.1371/journal.pone.0098682

**Published:** 2014-06-16

**Authors:** Martin Maripuu, Mikael Wikgren, Pontus Karling, Rolf Adolfsson, Karl-Fredrik Norrback

**Affiliations:** 1 Division of Psychiatry, Department of Clinical Sciences, Umeå University, Umeå, Sweden; 2 Division of Medicine, Department of Public Health and Clinical Medicine, Umeå University, Umeå, Sweden; University of Wuerzburg, Germany

## Abstract

**Background:**

Depression in unipolar and bipolar disorders is associated with hypothalamic-pituitary-adrenal-axis (HPA-axis) hyperactivity. Also, unipolar disorder has recently been shown to exhibit HPA-axis hypoactivity. We studied for the first time how HPA-axis hypo- and hyperactivity relate to depression and disease burden in bipolar disorder. We were interested in studying hypocortisolism; characterized by increased HPA-axis negative feedback sensitivity and lower basal cortisol levels together with the opposite HPA-axis regulatory pattern of hypercortisolism.

**Methods:**

This cross-sectional study includes 145 type 1 and 2 bipolar outpatients and 145 matched controls. A dexamethasone-suppression-test (DST) measures the negative feedback sensitivity and a weight-adjusted very-low-dose DST was employed, which is sensitive in identifying hypocortisolism and hypercortisolism. The 25th and 75th percentiles of control post-DST values were used as cut-offs identifying patients exhibiting relative hypo-, and hypercortisolism. Self-report questionnaires were employed: Beck-Depression-Inventory (BDI), Montgomery-Åsberg-Depression-Rating-Scale (MADRS-S), World-Health-Organization-Quality-of-Life-Assessment–100 and Global-Assessment-of-Functioning.

**Results:**

Patients exhibiting relative hypocortisolism expectedly exhibited lowered basal cortisol levels (p = 0.046). Patients exhibiting relative hypercortisolism expectedly exhibited elevated basal levels (p<0.001). Patients exhibiting relative hypocortisolism showed 1.9–2.0 (BDI, p = 0.017, MADRS-S, p = 0.37) and 6.0 (p<0.001) times increased frequencies of depression and low overall life quality compared with patients exhibiting mid post-DST values (eucortisolism). Adjusted Odds Ratios (OR:s) for depression ranged from 3.8–4.1 (BDI, p = 0.006, MADRS-S, p = 0.011) and was 23.4 (p<0.001) for life quality. Patients exhibiting relative hypercortisolism showed 1.9–2.4 (BDI, p = 0.017, MADRS-S, p = 0.003) and 4.7 (p<0.001) times higher frequencies of depression and low overall life quality compared with patients exhibiting eucortisolism. Adjusted OR:s for depression ranged from 2.2–2.7 (BDI, p = 0.068, MADRS-S, p = 0.045) and was 6.3 (p = 0.008) for life quality.

**Limitations:**

The cross-sectional design and lack of pre-established reference values of the DST employed.

**Conclusions:**

Relative hypocortisolism and relative hypercortisolism were associated with depression and lower life quality, providing novel insights into the detrimental role of stress in bipolar disorder.

## Introduction

Core features of bipolar disorder type 1 and 2 are depressive as well as manic and hypomanic episodes [1]. The significance of the depressive symptoms in bipolar disorder in terms of disease burden and time spent in depression has been highlighted during the last decade [2]–[8]. Dysregulation of the hypothalamic-pituitary-adrenal (HPA) axis has consistently been implicated in affective disorders. Stress, both acute and chronic, is recognized as an important etiologic factor of depression, that can affect the regulation of the HPA-axis, since the HPA-axis plays a crucial role in the neuroendocrine response to stress [9]. Stress has traditionally been associated with an increased activity of the HPA-axis, including increased cortisol levels and a decreased negative feedback sensitivity of the HPA-axis. This might partly explain, why in research on depressive symptomatology in unipolar and bipolar disorders, HPA-axis hyperactivity has been the main focus of attention and consistently reported [10]–[16].

However, Cushing's and Addison's disease are characterized by reduced and elevated cortisol levels, respectively and both exhibit high rates of depression which can be reversed with treatment aimed at normalizing the cortisol levels [17]–[20]. This supports the importance of HPA-axis homeostasis and that both hypo- and hyperactivity of the HPA-axis should be considered significant phenotypes, which ought to be compared with and understood relative to a normally regulated HPA-axis.

Hypoactivity of the HPA-axis has previously been observed, and suggested to develop out of chronic stress, in stress-related disorders such as PTSD, chronic fatigue syndrome, burn out and stress-related psychosomatic conditions, where an initial stage of HPA-axis hyperactivity eventually evolves into hypoactivity [21], [22]. The phenomenon of HPA-axis hypoactivity in stress-related disorders has increasingly been referred to as hypocortisolism, going back 10–15 years [22], [23]. Patients with affective disorders can also be expected to experience a high degree of chronic stress due to recurrent affective episodes and recently hypoactivity, in addition to hyperactivity, has been reported in patients suffering from unipolar depression [24]–[27]. To the best of our knowledge, the relationship between depressive symptoms and HPA-axis hypoactivity has however not been the focus of any studies in bipolar disorder. Also, there are still very few studies on unipolar and none on bipolar disorders that have adapted a homeostasis perspective when analyzing depressive symptoms in relation to the HPA-axis setting [24], [27].

Not a whole lot is known concerning the mechanistic underpinnings of hypocortisolism but it is known that mechanisms at different levels of the HPA-axis are potentially capable of producing decreased cortisol levels [22]. Even though the mechanisms behind hypocortisolism are largely unknown, researchers have been able to identify some core characteristics of the condition in stress-related disorders. These core characteristics are lowered cortisol levels during basal conditions, a reduced adrenocortiocal reactivity upon challenge and an increased negative feedback sensitivity of the HPA-axis [23]. Since no fixed, absolute cut-off values pertaining to these features have been adopted as part of the characterization of hypocortisolism, we will intermittently speak of *relative* hypocortisolism to indicate this fact. In a recent review it was also concluded that an increased negative feedback sensitivity of the HPA-axis as captured by the cortisol measure after employing low dose dexamethasone-suppression-tests (DST:s) was both the most common and the earliest characteristic of hypocortisolism [22].The DST consists of administration of an exogenous glucocorticoid in the form of dexamethasone, mimicking an elevated cortisol level which through negative feedback of the HPA-axis results in a lowering of the cortisol level. Hence the outcome of a DST, the post-DST cortisol value reflects the negative feedback sensitivity of the HPA-axis. However, since a low dose DST only moderately lowers the cortisol level, the outcome of the DST; the post-DST cortisol value should be recognized as a composite measure also reflecting, in part the basal cortisol level upon dexamethasone challenge.

By contrast, a decreased sensitivity of the negative feedback as well as elevated basal cortisol levels have in stress-related disorders, such as affective disorders, been considered characteristics of HPA-axis hyperactivity and have often been referred to as hypercortisolism [23], [28], [29]. We will intermittently speak of *relative* hypercortisolism to indicate that no fixed, absolute cut-off values pertaining to these features have been adopted as part of the characterization of hypercortisolism. An HPA-axis setting between the polar opposites of relative hypocortisolism and relative hypercortisolism will be denoted eucortisolism. Therefore, in order to evaluate the role of relative hypo- and hypercortisolism in bipolar disorder we chose to focus on evaluating the negative feedback sensitivity of the HPA-axis using a weight-adjusted very-low-dose DST.

The weight-adjusted-very-low-dose DST enables the identification of both an increased (a lower post-DST cortisol value) and a decreased (a higher post-DST cortisol value) negative feedback sensitivity of the HPA-axis and results in a graded response allowing for evaluation of the fine-tuning of the HPA-axis. This is in contrast to a conventional high dose DST that causes a “bottom” effect that makes it impossible to distinguish an increased negative feedback from a normal degree of cortisol suppression, post-dexamethasone challenge [30]. All DST:s regardless of the dexamethasone doses used however, have in common that they are mainly tests of the glucocorticoid receptor (GR) pathway sensitivity at the level of the pituitary since dexamethasone exhibits an increased affinity for the GR compared to the mineralocorticoid receptor (MR). The GR and MR are important in regulating both the level of cortisol and its effects throughout the body but the GR is thought to have a more central role during the stress response [31].

Quality of Life (QOL) and Global Functioning (GF) are core measures of the individual's well-being and overall level of functioning and are suitable measures to employ in order to capture the experienced burden of a disorder. There are well known associations between current depression and quality of life, however they are still thought of as different entities [32], [33] and the importance of evaluating QOL in both unipolar and bipolar disorders has been highlighted in recent reviews [34], [35]. There are no studies addressing QOL or GF in relation to the HPA-axis setting in bipolar disorder type 1 or 2. However, studies on such diverse conditions as subarachnoid hemorrhage, Cushing's disease and schizophrenia indicate an association between HPA-axis setting and QOL [36]–[38], and one study on unipolar disorder suggests an association between HPA-axis setting and GF [39].

We set out to investigate the relationships between depression and disease burden measured as QOL and GF in relation to relative hypo- and hypercortisolism in bipolar disorder. Bipolar patients with relative hypo- and hypercortisolism were identified as subjects exhibiting low- and high post-DST cortisol values, respectively. The study included 145 bipolar outpatients with varying degrees of depressive symptomatology, free of hypomania and mania, and 145 matched controls, making it one of the largest studies to date exploring the HPA-axis regulation in bipolar disorder.

## Methods

### Ethics Statement

The research was conducted according to the Helsinki declaration as revised 1989. All study participants gave written consent and the study was approved by the regional ethical review board of the medical faculty at Umeå University.

### Study participants

Outpatients with a bipolar type 1 or 2 diagnosis were considered for participation in the study, which is part of the multiple-outcome study; the Umeå Bipolar project. The patients were treated by a specialized outpatient affective unit at the Umeå University Hospital and enrolled continously between 1998–2007. All diagnoses were established by a senior psychiatrist according to DSM-IV criteria [40]. One-hundred-forty-five patients (89 bipolar type 1 and 56 bipolar type 2) fulfilled the in- and exclusion criteria and accepted participation. Exclusion criteria that could be mentioned: schizoaffective disorders, current hypomania or mania, neurologic disorders affecting the central nervous system including dementia and mental retardation, relatedness, use of corticosteroid medication as well as any other feature that would compromise the ability to fulfill the study protocol (e.g. not having Swedish as a mother tongue, severe visual or auditory handicaps).

The control sample consisted of 145 unrelated age- and sex-matched subjects from the Betula project [41]. The Betula project is a large multiple-outcome study focused at exploring memory, health and aging in the general population. All participants were randomly selected from the population registry of the same region as the patient sample (the Umeå region, northern Sweden) and have been shown to exhibit excellent representativity toward the general population [42]. The same exclusion criteria as for the patient sample were applied to the controls. Additionally however, current depression and known diagnoses of unipolar and bipolar disorders were excluded for.

### Questionnaires

Depressive symptomatology was evaluated with the self-report questionnaires of Beck Depression Inventory (BDI) [43] and Montgomery Åsberg Depression Rating Scale-Self assessment (MADRS-S) [44], [45]. Symptoms of anxiety were evaluated with the self-report questionnaires of Beck Anxiety Inventory (BAI) [44], [46] and Brief Scale for Anxiety-Self assessment (BSA-S) [44], [47]. BDI and BAI refer to last week whereas MADRS-S and BSA-S, which are sub-scales of the Comprehensive Psychopathological Rating Scale-Self -Affective (CPRS-S-A) [44], [48], refer to the last three days. GF was evaluated with the self-assessment version of the Global Assessment of Functioning (GAF) scale [40], [49]. It asks for the level of functioning last week and the highest level of functioning over three months last year. QOL was evaluated with the World Health Organization Quality of Life Assessment-100 (WHOQOL-100) [50] scale which is a self-report questionnaire concerning QOL during the last two weeks. The scale presents QOL as the overall QOL and general health and as 6 different domains of QOL: the physical, psychological, independence, social relationships, environmental and the spirituality domains. Concerning the depression and anxiety questionnaires, low scores indicate better health whereas for GAF and WHOQOL-100 high scores indicate better health.

### Dexamethasone suppression test

All patients and controls participated in a weight-adjusted very-low-dose DST. The DST has previously been employed in research on obesity but has also more recently been used by us in studies on affective disorder and general population samples [29], [51]. Study participants were instructed to ingest an individualized pre-measured solution containing dexamethasone at 11:00 p.m [29], [51]–[53]. The DST solution contained 3.5 µg of dexamethasone phosphate per kilogram of body weight (i.e. 175 µg and 280 µg for a person weighing 50 and 80 kg, respectively).The morning after ingestion, blood was drawn between 8:00 and 10:00 a.m. Randomly selected patient and control subgroups had their plasma analyzed for dexamethasone. All of them (24 patients and 95 controls) were positive for dexamethasone, demonstrating excellent compliance. In addition, basal morning cortisol was measured prior to the DST (also between 8:00 and 10:00 a.m.) in 73 patients and 84 controls.

### Statistical methods

Student's t-test was performed when testing for differences between two means and Pearson's chi-square test was employed when testing for differences in distribution of categorical data. Correlation analyses were performed using Pearson correlations. Analyses of covariance and logistic regression were applied for multivariate statistics. Multivariate statistics were performed unadjusted and in an adjusted model, adjusting for sex, age and diagnosis (bipolar type 1 or 2). We also tested for additional potential confounding of the following variables: body mass index, smoking or not, antidepressant medication or not, neuroleptic medication or not, sedative medication or not, mood stabilizer (lithium compounds or anti-epileptics) or not. The confounders listed above were included within the adjusted model (adjusting for sex, age and diagnosis) one at a time, the model was evaluated and the confounder was removed from the model before the next confounder was individually included within the adjusted model (adjusting for sex, age and diagnosis) and evaluated. When necessary, data were square root transformed to obtain a normal distribution. Established cut-offs, when existent, were employed when questionnaire scores were dichotomized. The following cut-offs were used: BDI>9 and MADRAS-S>11 were denoted as depression, and BAI>7 was denoted as anxiety. Concerning MADRS-S there appears to be a lack of consensus concerning the most suitable cut-off. The remaining questionnaire scores (BSA-S, WHOQOL-100, GAF) lacked established cut-offs and were therefore dichotomized, comparing the lowest quartile (denoted as low) with the rest. Probability values below 0.05 were considered significant. IBM SPSS Statistics 19 was used for all statistical analyses.

## Results

### Characteristics of study participants

Mean age of the patient and control samples were 47.8 and 49.3 years, respectively, with 61% females in both samples (Table 1). Within the bipolar sample, 61% were of type 1 and 39% of type 2. Forty-three percent of the patients were depressed according to BDI. For further description of patients and controls, see Table 1. Concerning the level of anxiety, GF and QOL exhibited by the patients, see Table 2.

**Table 1:** Study participant characteristics.

Patient and control subject characteristics	Patients (*n* = 145)	Control Subjects (*n* = 145)	*p*
Age, yrs (SD)	47.8 (13.9)	49.3 (11.3)	0.311
Gender, male/female (% male)	57/88 (39)	57/88 (39)	1.000
Basal cortisol, nmol/L (SD)^a^	457 (180)	394 (114)	0.011
Post-DST cortisol, nmol/L (SD)	362 (193)	322 (144)	0.042
BMI, kg/m^2^ (SD)^b^	26.3 (3.9)	25.3 (3.5)	0.030
Smoking, *n* (%)^c^	31 (21)	28 (19)	0.663
Low post-DST group, *n* (%) also denoted hypocortisolism^d^	37 (26)	36 (25)	1.000
Post-DST cortisol in the low post DST-group, nmol/L (SD)	152 (60)	148 (52)	0.770
High post-DST group, *n* (%) also denoted hypercortisolism^d^	48 (33)	35 (24)	0.119
Post-DST cortisol in the high post DST-group, nmol/L (SD)	581 (143)	516 (87)	0.012

Patient-specific characteristics		
Bipolar disorder type 1, *n* (%)	89 (61)	n/a
Bipolar disorder type 2, *n* (%)	56 (39)	n/a
Disease duration, yrs (SD)	25.1 (12.8)	n/a
Depression, BDI; *n* (%)	62 (43)	n/a
Depression, MADRS-S; *n* (%)	50 (35)	n/a
Antidepressant medication, *n* (%)^e^	42 (29)	n/a
Antipsychotic medication, *n* (%)^e^	40 (28)	n/a
Sedative medication, *n* (%)^e^	54 (37)	n/a
Mood stabilizing medication, *n* (%)^e^	109 (76)	n/a

BDI, Beck Depression Inventory; BMI, body mass index; DST, dexamethasone suppression test; MADRS-S, Montgomery Åsberg Depression Rating Scale – Self assessment; SD, standard deviation. All values are means unless otherwise specified. Student's t-test was performed when testing for differences between two means and Pearson's chi-square test was employed when testing for differences in distribution of categorical data.

a Patients, *n* = 73; control subjects, *n* = 84.

b Patients, *n* = 145; control subjects, *n* = 144.

c Current smoker.

d Post-DST cortisol groups were formed by using the 25th and 75th percentiles among the controls as cut-offs to divide both controls and patients into 3 groups. A low post DST cortisol value (subjects below the 25th percentile, also denoted the low post DST group) was used to identify subjects exhibiting relative hypocortisolism and a high post DST cortisol value (subjects above the 75th percentile, also denoted the high post DST group) was used to identify subjects exhibiting relative hypercortisolism. Subjects showing post DST cortisol values between the 25th and 75th percentiles were identified as subjects exhibiting eucortisolism.

e Number of patients on current medication.

**Table 2:** The relationships between relative hypo- and hypercortisolism and mean questionnaire scores among bipolar patients.

	HPA axis setting^a^				Hypo- vs. Eucortisolism		Hyper- vs. Eucortisolism		Hypo- and Hyper- vs. Eucortisolism	
	Hypocortisolism^a^ (*n* = 37)	Eucortisolism^a^ (*n* = 60)	Hypercortisolism^a^ (*n* = 48)	Total (*n* = 145)	*p*	*p* (adj.)	*p*	*p* (adj.)	*p*	*p* (adj.)
Depression										
BDI	12.6 (9.7)	7.9 (9.4)	13.5 (12.0)	11.0 (11.0)	0.013	0.004	0.004	0.038	0.001	0.004
MADRS-S	10.6 (8.3)	7.4 (7.6)	11.3 (8.2)	9.5 (8.1)	0.046	0.014	0.006	0.032	0.004	0.008
Anxiety										
BAI	9.9 (8.1)	7.8 (7.5)	10.1 (7.5)	9.2 (8.1)	0.170	0.133	0.097	0.353	0.064	0.155
BSA-S	10.6 (6.5)	8.5 (7.7)	11.2 (8.3)	9.9 (7.7)	0.071	0.040	0.044	0.210	0.019	0.054
Global Function^b^										
GAF current	72.3 (18.5)	81.4 (15.7)	76.7 (16.1)	77.6 (16.8)	0.040	0.023	0.137	0.519	0.037	0.081
GAF last year	71.5 (14.8)	78.6 (16.6)	70.8 (17.1)	74.3 (16.6)	0.045	0.024	0.020	0.165	0.009	0.019
Quality of Life^c^										
Overall QOL and general health	12.5 (4.0)	14.7 (3.0)	12.6 (3.8)	13.5 (3.6)	0.005	0.001	0.004	0.020	<0.001	0.001
Physical domain	13.0 (2.9)	14.5 (3.0)	13.1 (3.0)	13.7 (3.0)	0.018	0.009	0.042	0.157	0.009	0.020
Psychological domain	12.8 (3.1)	14.2 (2.6)	12.7 (3.0)	13.4 (2.9)	0.024	0.009	0.012	0.151	0.005	0.015
Independence domain	15.0 (2.6)	16.0 (2.1)	14.3 (2.9)	15.2 (2.6)	0.078	0.034	0.002	0.009	0.002	0.006
Social relationships domain	13.7 (2.8)	14.8 (2.4)	13.8 (2.6)	14.2 (2.6)	0.045	0.035	0.070	0.078	0.026	0.020
Environment domain	15.4 (1.8)	15.3 (1.8)	14.3 (2.2)	15.0 (2.0)	0.780	0.755	0.023	0.143	0.170	0.355
Spirituality domain	11.9 (4.4)	12.5 (3.9)	11.2 (3.7)	12.0 (4.0)	0.391	0.309	0.070	0.223	0.106	0.183

Adj, adjusted; BAI, Beck Anxiety Inventory; BSA-S, Brief Scale of Anxiety – Self assessment; BDI, Beck Depression Inventory; BMI, body mass index; DST, dexamethasone suppression test; GAF, Global Assessment of Functioning; MADRS-S, Montgomery Åsberg Depression Rating Scale – Self assessment; QOL, Quality of Life. All values are means with standard deviations within the following parentheses. Analyses of covariance were used when testing for adjusted differences between two means. The adjusted model contained the variables age, sex and diagnosis (bipolar type 1 or 2) in addition to cortisol group (low, mid, high). We also tested for additional potential confounding through individually including within the model the following variables: BMI, smoking, antidepressant medication, neuroleptic medication, sedative medication and medication with mood stabilizer (see statistical section). Significant adjusted analyses remained significant also after these additional potential confounders were added to the model except for a few instances where the cortisol variable showed a trend toward significance (p-values between 0.05 to 0.085): BSA-S, GAF current, the independence and social relationships domains (QOL) within the low vs. mid group analyses. In one of these additional potential confounding analyses however, when BMI was added to the adjusted model pertaining to low vs mid cortisol group comparisons with respect to the independence domain the analysis did not show significance nor trend (p = 0.153).

a Post-DST cortisol groups were formed by using the 25th and 75th percentiles among the controls as cut-offs to divide both controls and patients into 3 groups. A low post DST cortisol value (subjects below the 25th percentile) was used to identify subjects exhibiting relative hypocortisolism and a high post DST cortisol value (subjects above the 75th percentile) was used to identify subjects exhibiting relative hypercortisolism. Subjects showing post DST cortisol values between the 25th and 75th percentiles were identified as subjects exhibiting eucortisolism.

b In the GAF analyses *n* were as follows: low group, *n* = 37; mid group, *n* = 59, high group, *n* = 46 (*n* = total 142).

c In the Quality of Life analyses *n* were as follows: low group, *n* = 36; mid group, *n* = 60; high group, *n* = 48 (*n* = total 144).

### HPA-axis measures in patients and controls

A low post-DST cortisol value reflects an increased negative feedback sensitivity using a low dose DST and was used to identify subjects exhibiting relative hypocortisolism. A high post-DST cortisol value reflects a decreased negative feedback sensitivity and was used to identify subjects exhibiting relative hypercortisolism. Since no fixed, absolute post-DST cortisol cut-off values are part of the characterization of hypo-, or hypercortisolism and there are no established cut-offs for the weight-adjusted low-dose DST employed, we chose the 25th and 75th percentiles of the post-DST values among the controls as cut-offs to divide both controls and patients alike, into three groups. The post-DST cortisol groups were denoted the low (subjects below the 25th percentile, post DST cortisol <221.76 nmol/l), the mid (subjects between 25th and 75th percentiles) and the high post-DST cortisol group (subjects above the 75th percentile, post DST cortisol >408.75 nmol/l).

Within the patient sample, the low post-DST group exhibited significantly decreased basal morning cortisol compared to the mid and high group (381 vs. 480, p = 0.046, n = 73). This finding provide further confirmation that a low post-DST value employing a low dose DST does identify hypocortisolism in bipolar disorder since a decreased basal cortisol level is another characteristic of the phenomenon of hypocortisolism, in addition to an increased negative feedback sensitivity. The low post-DST patients also exhibited a significantly increased responsiveness to dexamethasone; reduction of the cortisol level between the basal and post-DST values (222 vs. 31, p<0.001, n = 73). An increased reduction between the basal and post-DST values is together with a low post-DST value measures that reflect an increased negative feedback sensitivity of the HPA-axis. The low post-DST group will also be referred to as the group exhibiting hypocortisolism or relative hypocortisolism.

Correspondingly, the high post-DST patient group showed significantly higher baseline morning cortisol levels (574 vs. 384, p<0.001, n = 73) providing further confirmation that a high post-DST cortisol value does identify hypercortisolism since an increased basal cortisol level is another characteristic of hypercortisolism, in addition to a decreased negative feedback sensitivity. The high post-DST group also showed a significantly decreased responsiveness to dexamethasone (−12 vs. 130, p<0.001, n = 73), compared to the low and mid cortisol group, which in addition to a high post-DST value captures the decreased negative feedback sensitivity exhibited by the patient group. The high post-DST group will also be referred to as the group exhibiting hypercortisolism or relative hypercortisolism, whereas the mid post-DST group will also be referred to as the eucortisolism group.

The same pattern was observed within the control sample, with the low post-DST group exhibiting a trend toward lower basal morning cortisol compared with the mid and high group (359 vs. 410, p = 0.056, n = 84), and a significantly increased responsiveness to dexamethasone (214 vs. 42, p<0.001, n = 84). Similarly, the high group showed significantly higher baseline morning cortisol (502 vs. 368, p<0.001, n = 84) and a lower degree of change between the basal and post-DST cortisol (2 vs. 120, p = 0.002, n = 84) compared to the mid and low group.

Comparing patients and controls with respect to the distribution of the three groups formed there were no significant differences in the proportions of individuals in the low and high groups (Table 1). Comparing the cortisol levels between patients and controls within the low and high groups separately, the patients in the high group exhibited significantly higher post-DST cortisol values compared with the controls in the high group, whereas for the low groups the difference was non-significant. When not taking the three groups into account, patients showed moderately but significantly elevated basal and post-DST cortisol values compared with controls (Table 1).

### Relative hypo- and hypercortisolism are associated with depression

**Figure 1:**
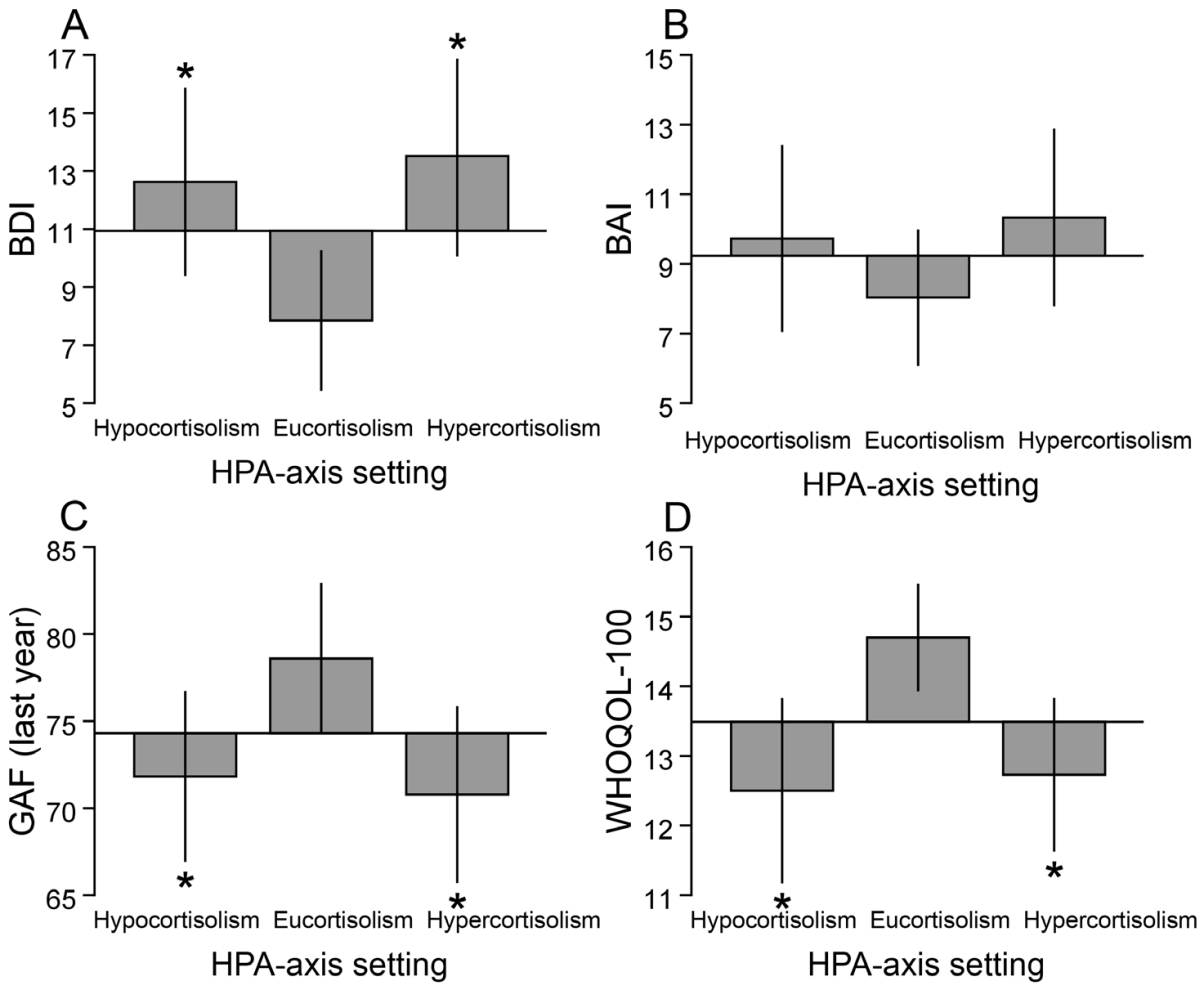
Illustration of relative hypo- and hypercortisolism in relation to depression, anxiety, global functioning and life quality. Post-DST cortisol groups were formed by using the 25th and 75th percentiles among the controls as cut-offs to divide both controls and patients into 3 groups. A low post DST cortisol value (subjects below the 25th percentile) was used to identify subjects exhibiting relative hypocortisolism and a high post DST cortisol value (subjects above the 75th percentile) was used to identify subjects exhibiting relative hypercortisolism. Subjects showing post DST cortisol values between the 25th and 75th percentiles were identified as subjects exhibiting eucortisolism. The bars illustrate the differences in mean questionnaire scores relative to the mean of the whole bipolar patient sample which is indicated by the base-line. A bar marked with an asterisk denotes a significant difference, evaluated using Student's t-test, between relative hypocortisolism or relative hypercortisolism and the reference group exhibiting eucortisolism. The error bars represent standard errors. BAI, Beck Anxiety Inventory; BDI, Beck Depression Inventory; DST, Dexamethasone Suppression Test; GAF, Global Assessment of Functioning (best period of three months last year); QOL, Overall Quality of Life and general health. *p<0.05.

Seventy-four percent of the patients suffering from depression, based on BDI, exhibited relative hypo- or hypercortisolism (76% based on MADRS-S). Relative hypocortisolism was associated with a higher frequency of depression (54% vs. 28%, p = 0.017, n = 97, based on BDI; 41% vs. 20%, p = 0.037, n = 97, MADRS-S-based), higher mean values of depressive symptoms and higher odds ratios (OR:s) of being depressed compared with eucortisolism (the mid group) in adjusted and unadjusted analyses (Table 2, 3, Figure 1).

**Table 3:** The relationships between relative hypo- and hypercortisolism and depression as well as low global functioning and quality of life in bipolar disorder.

	Hypocortisolism vs. Eucortisolism^a^ (n = 97)				Hypercortisolism vs. Eucortisolism^a^ (n = 108)				Hypo- and Hypercortisolism vs. Eucortisolism^a^ (n = 145)			
	unadjusted		adjusted^b^		unadjusted		adjusted^b^		unadjusted		adjusted^b^	
	OR (CI)	*p*	OR (CI)	*p*	OR (CI)	*p*	OR (CI)	*p*	OR (CI)	*p*	OR (CI)	*p*
Depression												
BDI	2.98 (1.26–7.01)	0.013	3.76 (1.46–9.66)	0.006	2.75 (1.24–6.10)	0.013	2.21 (0.94–5.19)	0.068	2.85 (1.41–5.76)	0.004	2.77 (1.32–5.82)	0.007
MADRS-S	2.73 (1.10–6.79)	0.031	4.12 (1.39–12.25)	0.011	3.68 (1.57–8.60)	0.003	2.68 (1.02–7.03)	0.045	3.23 (1.51–6.94)	0.003	3.23 (1.38–7.58)	0.001
Anxiety												
BAI	1.29 (0.57–2.93)	0.543	1.34 (0.56–3.19)	0.514	1.33 (0.62–2.84)	0.465	1.00 (0.44–2.27)	0.994	1.31 (0.68–3.33)	0.423	1.13 (0.57–2.26)	0.723
BSA-S	2.58 (0.93–7.21)	0.070	2.76 (0.96–7.91)	0.059	2.93 (1.12–7.68)	0.029	2.49 (0.91–6.81)	0.076	2.77 (1.25–6.15)	0.012	2.58 (1.13–5.89)	0.025
Global Function^c^												
GAF current	1.51 (0.57–3.96)	0.406	1.72 (0.61–4.81)	0.303	1.38 (0.55–3.45)	0.487	0.95 (0.35–2.61)	0.922	1.44 (0.65–3.20)	0.375	1.24 (0.54–2.85)	0.619
GAF last year	2.08 (0.78–5.52)	0.145	2.46 (0.85–7.09)	0.096	2.61 (1.05–6.50)	0.039	1.72 (0.60–4.92)	0.311	2.36 (1.04–5.37)	0.040	2.09 (0.87–5.02)	0.101
Quality of Life^d^												
Overall QOL	10.00 (2.98–33.59)	<0.001	23.44 (4.70–116.91)	<0.001	7.00 (2.15–22.75)	0.001	6.31 (1.61–24.79)	0.008	8.19 (2.71–24.77)	<0.001	10.48 (3.07–35.82)	<0.001
Physical domain	1.96 (0.75–5.14)	0.172	2.35 (0.81–6.81)	0.116	2.03 (0.83–4.95)	0.122	1.52 (0.56–4.09)	0.410	2.00 (0.90–4.45)	0.091	1.78 (0.76–4.18)	0.186
Psychological domain	4.28 (1.51–12.12)	0.006	5.83 (1.81–18.77)	0.003	3.79 (1.41–10.20)	0.008	2.06 (0.63–6.69)	0.230	3.99 (1.61–9.89)	0.003	3.83 (1.43–10.21)	0.007
Independence domain	4.50 (1.51–13.40)	0.007	6.54 (1.86–23.00)	0.003	4.94 (1.76–13.83)	0.002	3.97 (1.21–13.06)	0.023	4.75 (1.83–12.34)	0.001	4.74 (1.69–13.24)	0.003
Social relationships domain	3.61 (1.36–9.56)	0.010	4.08 (1.46–11.40)	0.007	1.89 (0.72–4.95)	0.196	1.77 (0.62–5.03)	0.284	2.54 (1.09–5.92)	0.031	2.81 (1.15–6.86)	0.023
Environment domain	0.80 (0.27–2.36)	0.686	0.81 (0.27–2.40)	0.702	2.62 (1.11–6.18)	0.028	1.88 (0.74–4.73)	0.183	1.70 (0.77–3.72)	0.189	1.44 (0.63–3.28)	0.387
Spirituality domain	1.14 (0.42–3.13)	0.795	1.23 (0.42–3.62)	0.712	1.19 (0.47–3.00)	0.713	0.89 (0.32–2.49)	0.825	1.17 (0.52–2.64)	0.706	1.02 (0.43–2.45)	0.962

BAI, Beck Anxiety Inventory; BSA-S, Brief Scale for Anxiety – Self assessment; BDI, Beck Depression Inventory; BMI, body mass index; CI, 95% confidence interval; DST, dexamethasone suppression test; GAF, Global Assessment of Functioning; MADRS-S, Montgomery Åsberg Depression Rating Scale – Self assessment; OR, odds ratio; QOL, Quality of Life.

The table describes the OR:s of the high or low post-DST groups as compared to the mid-group (the reference group) for exhibiting depression, anxiety, low quality of life and low global functioning. The logistic regression analyses were performed unadjusted and in a model adjusting for age, sex and diagnosis (bipolar type 1 or 2). The outcome variables of the logistic regression analyses were the questionnaire scores dichotomized according to established cut-offs when present (BDI, MADRS-S, BAI, See the statistical methods paragraph). The remaining questionnaire scores (BSA-S, GAF WHOQOL-100) which lacked established cut-offs were dichotomized comparing the lowest quartile with the rest. We also tested for additional potential confounding through individually including within the model the following variables: BMI, smoking, antidepressant medication, neuroleptic medication, sedative medication and medication with mood stabilizer (see statistical section). Significant adjusted analyses remained significant also after these additional potential confounders were added to the model except for one instance when BMI was added to the high vs. mid analyses with respect to MADRS-S where the cortisol group variable showed a trend toward significance (p = 0.054).

a Post-DST cortisol groups were formed by using the 25th and 75th percentiles among the controls as cut-offs to divide both controls and patients into 3 groups. A low post DST cortisol value (subjects below the 25th percentile) was used to identify subjects exhibiting relative hypocortisolism and a high post DST cortisol value (subjects above the 75th percentile) was used to identify subjects exhibiting relative hypercortisolism. Subjects showing post DST cortisol values between the 25th and 75th percentiles were identified as subjects exhibiting eucortisolism.

b Adjusted for sex, age and diagnosis (bipolar type 1 or 2).

c In the GAF analyses *n* were as follows: low vs. mid cortisol group, *n* = 96; high vs. mid cortisol group, *n* = 105; low and high vs. mid cortisol group, *n* = 142.

d In the Quality of Life analyses *n* were as follows: low vs. mid cortisol group, *n* = 96; high vs. mid cortisol group, *n* = 108; low and high vs. mid cortisol group, *n* = 144.

Similarly, patients in the group exhibiting relative hypercortisolism showed a higher frequency of depression (52% vs. 28%, p = 0.017, n = 108, based on BDI; 48% vs. 20%, p = 0.003, n = 108, MADRS-S-based), higher mean values of depressive symptoms and higher OR:s of being depressed compared with the eucortisolemic group (Table 2, 3, Figure 1). The only analysis that did not reach significance was the BDI-based adjusted logistic regression analysis which showed trend (p = 0.068, Table 3).

The relationships between anxiety and relative hypo- and hypercortisolism were weaker than those between depression and the HPA-axis setting and were generally non-significant. (Table 2, 3, Figure 1).

### Relative hypo- and hypercortisolism are associated with lower quality of life

Eighty-nine percent of the patients with low overall QOL exhibited relative hypo- or hypercortisolism. Patients in the group exhibiting relative hypocortisolism had a 6.0 times higher frequency of low overall QOL compared with the group exhibiting eucortisolism (42% vs 7%, p<0.001, n = 96). In adjusted analyses, they exhibited lower mean scores on overall QOL and on all domains except for the environment and spirituality domains when compared with the eucortisolism group (Table 2). They also exhibited higher OR:s of having a low QOL with respect to overall QOL and all domains in adjusted analyses except for the physical, environment and spirituality domains compared with the group exhibiting eucortisolism (Table 3). The patient group exhibiting relative hypercortisolism had a 4.7 times higher frequency of low overall QOL compared with the group exhibiting eucortisolism (33% vs 7%, p<0.001, n = 108). In unadjusted analyses, they exhibited lower mean scores on overall QOL and on all domains except for social relationships and spirituality compared with the eucortisolism group (Table 2). They also exhibited higher OR:s of having a low QOL with respect to overall QOL and all domains except the physical, social relationship and spirituality domains in unadjusted analyses (Table 3). When adjusted analyses were performed significant relationships remained between relative hypercortisolism and overall QOL and the independence domain concerning both mean score comparisons and OR analyses (Table 2, 3, Figure 1).

### The relationships between relative hypo- and hypercortisolism and global functioning

The patient hypocortisolism group showed lower mean scores on GAF current and GAF last year compared with the eucortisolism group in adjusted analyses (Table 2, Figure 1). The patient hypercortisolism group only showed lower GAF last year scores compared with the eucortisolism group in unadjusted analyses (Table 2, Figure 1). Neither relative hypo- nor hypercortisolism significantly predicted low GF when the GAF scores were compressed into a dichotomy within the logistic regression analyses (Table 3).

### Eucortisolism is associated with less depression and higher quality of life

We compared the patient group exhibiting eucortisolism with the patient hypo- and hypercortisolism groups added together. As expected, the group exhibiting eucortisolism showed a lower degree of depression and higher QOL with respect to the overall measure and several specific domains (Table 2, 3). Eucortisolism was associated with higher mean scores on the GAF instrument but did not predict high GF when the GAF scores were dichotomized within the logistic regression analyses (Table 2, 3). Anxiety showed a trend to be lower in the group exhibiting eucortisolism, but differences were mostly non-significant (Table 2, 3). We also evaluated whether the magnitude of the deviation from an “ideal” post-DST cortisol value was positively correlated with more symptoms, lower QOL and GF. To that effect, we chose the median post-DST cortisol value within the control group as an “ideal” value (308 nmol/l) and the absolute difference from that value was calculated for each patient. The absolute deviation was correlated with depressive symptoms, QOL (except for social relationships), GAF last year and anxiety measured using BSA-S (Table 4).

**Table 4:** Correlations between questionnaire scores and the absolute post-DST cortisol deviation among bipolar patients.

	Pearson correlations (*n* = 145)	
	*r*	*p*
Depression		
BDI	0.228	0.006
MADRS-S	0.229	0.006
Anxiety		
BAI	0.129	0.122
BSA-S	0.170	0.041
Global Functioning^a^		
GAF current	−0.145	0.085
GAF last year	−0.228	0.006
Quality of Life^b^		
Overall QOL	−0.229	0.006
Physical domain	−0.202	0.015
Psychological domain	−0.241	0.004
Independence domain	−0.291	<0.001
Social relationships domain	−0.116	0.167
Environment domain	−0.215	0.010
Spirituality domain	−0.170	0.041

BAI, Beck Anxiety Inventory; BSA-S, Brief Scale for Anxiety – Self assessment; BDI, Beck Depression Inventory; DST, dexamethasone suppression test; GAF, Global Assessment of Functioning; MADRS-S, Montgomery Åsberg Depression Rating Scale – Self assessment; QOL, Quality of Life. We aimed to evaluate whether the magnitude of the deviation from an “ideal” post-DST cortisol value within the patient sample was positively correlated with more symptoms, lower global functioning and QOL (for a more detailed explanation see the results and discussion sections). To that effect, we chose the median post-DST cortisol value within the control group as an “ideal” value (308 nmol/l) and the absolute post-DST cortisol deviation from that value was calculated for each patient. Bivariate correlations were performed using Pearson correlation.

a Global Functioning analyses, *n* = 142.

b Quality of Life analyses, *n* = 144.

## Discussion

The main aim was to investigate the relationships between hypo- and hypercortisolism and depressive symptoms as well as disease burden, measured as QOL and GF, in bipolar disorder. Bipolar patients with relative hypo- and hypercortisolism were identified as subjects exhibiting low- and high post-DST cortisol values, respectively, employing a low dose DST.

We found that relative hypocortisolism was associated with depressive symptoms in bipolar disorder compared with eucortisolism. We also found that hypocortisolism was associated with lower QOL and GF compared with eucortisolism. To the best of our knowledge, these results are novel findings. Relative hypercortisolism compared with eucortisolism similarly exhibited associations with depressive symptoms and lowered QOL, with the latter also being a novel finding.

The finding that hypocortisolism was associated with depressive symptoms is in line with recent studies describing an association between hypocortisolism and unipolar depression [24]–[27]. The finding that hypercortisolism was associated with depressive symptoms in bipolar disorder corroborates previous reports [11]–[14], [16].

It is clear that without the homeostasis perspective which was adapted, that considered both lowered and increased levels of cortisol as potential risk phenotypes compared with eucortisolism, the findings above would have been partly or completely obscured. We believe this could be the case in some earlier studies on affective disorders which only considered either hypo- or hyperactivity of the HPA-axis as potentially clinically relevant.

QOL was lower in both the groups exhibiting hypo- and hypercortisolism compared to the group exhibiting eucortisolism. The effect sizes were large with 89% of the patients with low QOL exhibiting hypo- or hypercortisolism, and low as well as high post-DST cortisol levels predicted low overall QOL with adjusted OR:s of 23.4 and 6.3, respectively. This suggests that QOL and the HPA-axis setting are intimately related. QOL is known to be impaired both in depressed and euthymic bipolar patients and there is a need of treatment regimens that are efficient in reducing depressive symptoms as well as improving QOL [34], [54]. Therefore, it is potentially of great interest that when the degree of depression measured as BDI scores was added to the adjusted model (see statistical methods), both relative hypo- and hypercortisolism remained significant predictors of low overall QOL (OR = 26.1, p = 0.001, n = 97 and OR = 14.8, p = 0.013, n = 108, respectively). The same analyses based on MADRS-S scores were also significant (OR = 33.7, p = 0.001, n = 97 and OR = 11.3, p = 0.009, n = 108 for hypo- and hypercortisolism, respectively). These results indicate the existence of a depression-independent pathway between the HPA-axis setting and QOL. To the best of our knowledge, there are no studies on bipolar disorder investigating the relationship between HPA-axis regulation and QOL. More studies are thus needed investigating these potential causal relations which will provide insight into what effects treatment of HPA-axis dysregulation in bipolar disorder will have upon not only depression but also QOL.

Analyses of GF showed lower average GAF scores within the group exhibiting hypocortisolism compared with the group exhibiting eucortisolism. This is partly supported by a study on unipolar depression showing that patients exhibiting hypercortisolism had higher GAF scores than the rest, which consisted of unknown fractions of patients exhibiting eucortisolism and hypocortisolism [39]. There are no other comparable studies that can shed light on this association and the results are in need of replication and a more detailed investigation.

From a cross-sectional design it is impossible to distinguish between causes and effects. Thus the causal directions between HPA-axis setting, depression and well-being cannot be elucidated. However, in addition to studies on affective disorders, studies focusing on corticosteroid medication, Cushing's and Addison's disorder in relation to depression also indicate that increased as well as decreased cortisol levels could be causally involved in depression [17], [18], [55]–[59]. The opposite could however also pertain, since being currently depressed could contribute causally to hypercortisolism, since suffering from depression is a significant stressor and increased stress can result in increased cortisol levels. It is also possible that depression could cause hypocortisolism in bipolar disorder, since recurring depressive episodes and social consequences of these symptoms could become chronic stressors and a recent meta-review concluded that hypocortisolism most commonly seems to develop out of chronic stress [21]. In support of such a possibility could be taken our finding that the hypocortisolism group displayed a longer mean disease duration compared with the hypercortisolism group (27.2 vs. 21.7 years, p = 0.039, n = 85) and bipolar patients are known to suffer from affective symptoms for a large portion of the time (around 50%), despite appropriate treatment [3], [5]. Other connected, interesting questions that should be addressed in future studies are whether hypocortisolism, compared to hypercortisolism, is a more stable, chronic state in bipolar disorder once developed as well as whether hypocortisolism could act as a maintenance factor of chronic depression and residual depressive symptoms; both of which are prevalent in bipolar disorder [2], [3], [5], [7], [60], [61].

Aside from the issue of the potential causality relations between the HPA-axis setting and depression as well as quality of life, what can be said about the regulation of the HPA-axis in bipolar disorder? One initial aspect worthy of mentioning is that most of the patients cannot be said to exhibit abnormally decreased or increased post-DST cortisol levels. At least not in the sense, that their cortisol values did not overlap with those of the controls from the general population. It is however, important to remember that the general population sample does not only consist of subjects who are healthy without exposure to significant stressors and stress-related pathophysiological changes. Also, the results showed V-shaped relationships between the post-DST cortisol levels and depression as well as with QOL and GF (Table 4). This could be interpreted to support that bipolar patients exhibit vulnerability toward relatively small deviations of the HPA-axis setting and require an optimal functioning of the stress systems. It could also mean that, within the context of the stress-load of each individual bipolar patient, that a significant fraction of the patients actually do exhibit abnormally decreased or increased post-DST cortisol levels, in the sense that their cortisol values do not reflect a well functioning stress-response.

We will below focus on the phenomenon of hypocortisolism reported to exist in different stress-related conditions, such as PTSD, chronic fatigue syndrome, burn out, unipolar depression and stress-related psychosomatic conditions [22], [23] since less is known about it as compared to hypercortisolism. Although the mechanistic underpinnings of hypocortisolism are largely unknown a recent review has been able to identify some core characteristics of the condition in these stress-related disorders. These features of the condition were then used as the foundation of a criteria-based usage of the term ‘hypocortisolism’. These characteristics are: a reduced adrenocortical secretion during basal conditions at least temporarily during the circadian cycle, or a reduced adrenocortical reactivity upon challenge or an increased negative feedback sensitivity of the HPA axis [23]. Remember also, that no fixed, absolute values pertaining to these features are as yet part of the characterization of hypocortisolism and hence to speak of relative hypocortisolism could be considered appropriate (the same applies for the characterization of hypercortisolism). In a second review it was also concluded that an increased negative feedback sensitivity of the HPA-axis as captured by the post-DST cortisol measure employing low dose DST:s was both the most common and the earliest characteristic of hypocortisolism [22]. One possible reason why a measurement of the basal cortisol level appears to be a less reliable, informative indicator of the HPA-axis setting, could be the large degree of intra-individual variability observed between the circadian cycles, from day to day. Therefore, we chose to focus on evaluating the negative feedback sensitivity of the HPA-axis using a weight-adjusted very-low-dose DST and bipolar patients with relative hypo- and hypercortisolism were identified as subjects exhibiting low- and high post-DST cortisol values, respectively.

Based on the proposed core characteristics of the HPA-axis changes associated with hypocortisolism, we wanted to examine the relationships between the HPA-axis regulatory measures within the present study, in our bipolar patient and control samples. Subjects with low post-DST values, were also found to exhibit low basal state cortisol values and increased reductions between the basal and post-DST cortisol values, which similarly to the post-DST cortisol value is a measure reflecting the negative feedback sensitivity. This HPA-axis regulatory pattern was both observed among the patients and the controls representative of the general population. These measures are all in line with the core characteristics of hypocortisolism identified in the above mentioned review [23]. Patients and controls with high post-DST values on the other hand, exhibited the opposite pattern, since they showed high basal state cortisol values and decreased reductions between the basal and post-DST cortisol values. This HPA-axis regulatory pattern is the expected finding in subjects exhibiting hypercortisolism in affective disorder and in general population samples [23], [28], [29]. We have also previously reported on these two HPA-axis regulatory patterns in 81 unipolar depression patients and in a control sample representative of the general population consisting of 253 subjects (the controls however, partly overlap with the controls included within the present study) employing exactly the same DST and design in dividing the samples into groups denoted to exhibit relative hypo- and hypercortisolism [29]. From the findings that these two patterns, exhibited such a high degree of similarity between bipolar and unipolar patients as well as controls representative of the general population, we believe the conclusion should be drawn that one should look for a partly but not a completely bipolar-disorder-specific explanatory model for the HPA-axis regulatory changes observed in bipolar disorder.

The term hypocortisolism could be interpreted to imply deficient levels, i.e. deficient effects of cortisol. We however, want to make clear that when ascribing hypocortisolism to a subject it is an ascription that pertains to the level of cortisol and we are not claiming to know that the subject in question exhibits deficient cortisol effects. The subjects exhibiting hypocortisolism within the study showed an increased negative feedback sensitivity of the HPA-axis and hence an increased cortisol effect at the pituitary level. This localized increased cortisol sensitivity however, does not provide us with information concerning the sensitivity toward cortisol in other tissues, such as: liver, adipose tissue, the immune system or areas of the brain other than the pituitary. But this is not to say that it is not a real and interesting possibility that hypocortisolism could be characterized by cortisol deficiency either locally in some tissues or globally throughout the body, with the exception of the pituitary. It is however, also a possibility that other tissues in addition to the pituitary exhibit an increased cortisol sensitivity, thereby counteracting the decreased cortisol levels resulting in normal or even increased cortisol tissue effects. We hold the same attitudes as above, also toward the question whether hypercortisolism in stress-related disorders, is characterized by excessive cortisol effects or not.

In addition to factors determining the tissue effects of a given cortisol level there are also a multitude of factors influencing the level of cortisol. Mechanisms at several levels of the HPA axis are potentially capable of producing decreased or increased cortisol levels, respectively [21]. Again focusing on hypocortisolism, it is not currently believed that there is a singular mechanistic background for the phenomenon which is shared across all different stress-related conditions or shared among all individuals exhibiting hypocortisolism within the same disorder but rather that there could be multiple developmental pathways toward the state of hypocortisolism [22]. In elucidating the etiology of hypo- and hypercortisolism a lot of research will most likely be necessary, since it has been shown that the regulation of the HPA-axis is complex and influenced by multiple interacting factors such as: exposure to different types of stressors, person-dependent factors (how stressors are perceived, previous experiences, coping strategies etc) and heritable factors. Concerning heritability, it has for example been shown that polymorphisms within the genes involved in the functioning of the HPA-axis (e.g. genes encoding the GR, MR, FKBP5, CRH-R1) can influence the HPA-axis setting, including the negative feedback sensitivity [21], [31].

Despite the large number of candidate determinants of hypo- and hypercortisolism in bipolar disorder we still feel that a warranted point of departure for future research is offered by the theory that hypocortisolism in many cases develops out of long-term chronic stress, where an initial stage of a hyperactive HPA-axis eventually evolves into a hypoactive one. In applying this theory it is proposed that hypercortisolism most likely reflects a current increased stress-load compared to eucortisolism whereas hypocortisolism might not reflect the current stress-load of the subject but instead potentially taps the exposure-history toward stressors, and perhaps especially toward chronic stressors. Another potential research direction of interest, would be to investigate whether the quality of depression in bipolar disorder could be one major determinant of the HPA-axis changes observed, since it has been reported that depression with atypical and melancholic features differ with respect to the cortisol status in unipolar depression [62].

There is a great need of improvement concerning the treatment strategies of depression in bipolar disorder. Judd's et al long-term longitudinal studies have shown that bipolar type 1 and 2 patients spend 30–50% of the time suffering from depressive symptoms, despite modern treatment [2], [3], [5]. Both hypo- and hypercortisolism constitute interesting targets for novel treatment strategies. Although the research still remains to be performed aimed at understanding and determining the cortisol net-effects of both hypo- and hypercortisolism in bipolar disorder, there are studies indicating that both treatment regimens that lower as well as elevate the cortisol levels can have positive effects on depressive symptomatology. Lithium augmentation has in depressive disorder been shown to increase HPA-axis activity and further, this increase has been associated with short-term treatment efficiency [63], [64]. There is a pilot study showing promising results with low-dose prednisolone treatment of treatment-resistant depressive disorder patients exhibiting hypocortisolism [65]. Also, there are studies where corticosteroid treatment or inhibition of such effects has been effective, but the results were not evaluated in relation to hypo- and hypercortisolism. Such studies include: treatment of unipolar depression with hydrocortisone [66], treatment of bipolar and unipolar depression with dexamethasone [67], [68], glucocorticoid receptor antagonists [69] and inhibitors of glucocorticoid synthesis [69].

The HPA-axis setting could also become part of the treatment guidelines for affective disorders when choosing between already commonly used treatment regimens. Drugs used in the psychiatric clinical setting have been reported to differentially affect the regulation of the HPA-axis [70]. Hence studies systematically evaluating the clinical responses to these drugs within the context of relative hypo- and hypercortisolism are warranted. If the specific changes of the downstream effects of cortisol associated with hypo- and hypercortisolism that potentially exert the observed influence on mood and QOL could be identified it would be of great interest for future efforts in the development of new treatment strategies. This is beyond the scope of this study; however, loss of control of inflammation constitutes one such intriguing downstream pathway. Cortisol is an important modulatory factor of the immune system and recent years' research has produced a growing pool of evidence linking depression with inflammation [62], [71], [72]. In support of this line of thinking we recently showed that relative hypocortisolism in the general population was associated with a heightened level of inflammation [29].

The main limitations of the study was the cross-sectional design, which does not allow for drawing causal conclusions and the lack of pre-established reference values of the weight-adjusted very-low-dose DST employed.

In conclusion, this study clearly shows that relative hypo- and hypercortisolism are associated with more depressive symptoms and lower QOL compared with eucortisolism in individuals suffering from bipolar disorder. An interesting question for future research is whether hypo- and hypercortisolism could be prevented through early intervention, possibly through decreasing the number or severity of the affective episodes and hence the accumulated stress load upon the individual. We believe these results to be of great importance, offering new insights into the detrimental role of stress in bipolar disorder.
